# Digital Biometrics in Predicting Risk for Obstructive Sleep Apnea and Hypertension: Decentralized, Prospective Cohort Study

**DOI:** 10.2196/77641

**Published:** 2025-12-03

**Authors:** Natalia Orendain, Samantha Spierling Bagsic, Edward Ramos, Jay Pandit, Robert L Owens, Stuti Jaiswal

**Affiliations:** 1 Department of Research & Development, Scripps Health, La Jolla, CA, United States; 2 Department of Translational Medicine, Scripps Research Institute, 3344 N Torrey Pines Ct. Ste 300, La Jolla, CA, 92037, United States, +1 858-784-1000; 3 Division of Pulmonary, Sleep, and Critical Care, University of California San Diego School of Medicine, La Jolla, CA, United States

**Keywords:** digital health, sleep health, OSA, hypertension, wearable, cardiovascular disease, obstructive sleep apnea

## Abstract

**Background:**

Sleep is an important component of human health and can be measured longitudinally using digital activity trackers. Further, decentralized digital research has the potential to provide a real-world picture of sleep in large populations.

**Objective:**

This study examined whether longitudinal sleep patterns from activity trackers could predict risk of obstructive sleep apnea (OSA) and hypertension, as defined by the Berlin questionnaire and self-report, respectively.

**Methods:**

We recruited adults aged ≥18 years nationwide to join our sleep-focused smartphone-based study, called the Research Framework for Exploring Sleep Health. Our sample of 391 adults predominantly comprised women (68%, 247/364) with a mean age of 48 (SD 13.62) years. Participants were asked to fill out health-related surveys, including the Berlin questionnaire and the Horne-Ostberg questionnaire for chronotype. Participants were asked to link their own activity tracker to the app to collect longitudinal sleep data.

**Results:**

We analyzed sleep data from 391 participants; the cohort was predominantly White (65%, 231/353) followed by multiracial (17%, 61/353) and Hispanic or Latino (6.5%, 23/353) participants. Collinearity testing showed that OSA risk and self-reported hypertension could be considered independently. Holding BMI at a fixed value, the odds of having high OSA risk increased by 159% for every 1-hour increase in weekday sleep variability (odds ratio [OR] 2.592, 95% CI 1.613-4.400; *P*<.001), and the odds of high OSA risk increased by 93% for each 1-hour increase in weekend sleep variability (OR 1.928, 95% CI 1.197-3.094; *P=*.01). The odds of having high OSA risk increased by 22% for each unit (kg/m^2^) increase in BMI, holding both weekday and weekend sleep at separate fixed values (OR 1.217, 95% CI 1.153-1.293; *P*<.001). Controlling for age, sex, and BMI, the odds of endorsing hypertension increased by 71% for every 1-hour increase in weekday sleep variability (OR 1.712, 95% CI 1.062-2.917; *P=*.03). Conversely, for weekend sleep, the odds of endorsing hypertension increased by 43% for a 1-hour increase in weekend sleep variability (OR 1.432, 95% CI 1.062-1.928; *P=*.04). Increased sleep variability predicted a high risk for both OSA and hypertension in this decentralized cohort, when using data from the Berlin questionnaire.

**Conclusions:**

Our study demonstrates the utility of decentralized digital health studies in sleep research. It highlights the potential of activity trackers to predict risk for OSA and hypertension without requiring other patient information or assessment. Sleep variability is gaining increasing importance in the context of sleep health. Digital devices have the potential to help individuals assess their risk for certain disorders.

## Introduction

Recently, smartphone-based studies that use personal sensor technology have demonstrated the effectiveness in tracking, predicting, and intervening on human health outcomes [1,2]. However, studies in this space are still emerging, and much remains to be learned regarding the role of mobile technologies in disease prediction.

Healthy sleep habits are essential in maintaining human health and well-being. Inadequate or impaired sleep is associated with many diseases and disorders, such as cardiovascular disease (CVD), diabetes, and mental health disorders. While sleep duration is relatively well studied, other aspects of sleep are not, particularly those that require longitudinal measurements. For example, sleep variability refers to how regular a patient’s sleep is from night to night [3], and it is often measured as the standard deviation of their mean sleep duration. Another example is social jet lag [4], which is a form of circadian misalignment that compares how much sleep a person obtains on working days compared to nonworking days. Digital activity trackers (DATs) have been validated for basic measurements of sleep [5] and offer an opportunity to obtain daily sleep information over long periods of time, as demonstrated by recent studies such as the SleepHealth Mobile App Study [6].

Based on the premise that longitudinally collected sleep data could provide clinically meaningful insights into human health and could ultimately help inform patients and providers regarding sleep and health risks, we developed the Research Framework for Exploring Sleep Health (REFRESH) study to collect data from DATs while also collecting survey data related to health outcomes. This prospective, observational study employs a decentralized trial design that uses mobile recruitment and a study-specific smartphone app for data collection. Participants were invited to link a DAT to the study if they owned one, thus allowing for sleep data collection. The overarching hypothesis of this study was that sleep data from DAT devices could help identify an individual’s risk for sleep-related diseases, as well as other diseases that have been associated with inadequate sleep. Here we focus on disorders associated with CVD. Our study adds to the growing literature in this space with validated sleep and mental health questionnaires; intermittent assessments of mood, stress, and subjective sleep; and also an a priori focus on collecting data in the context of social jet lag and other weekend to weekday sleep differences.

First, obstructive sleep apnea [7] (OSA) is an important, highly prevalent [8] form of sleep-disordered breathing that occurs when the upper airway becomes blocked, thus leading to short periods of hypoxemia, elevated heart rate, and repetitive arousals from sleep; ultimately, vascular and cellular damage ensues. Twenty years of research associates OSA and hypertension in a dose-dependent manner, with OSA predicting the development of new-onset hypertension [9]. In addition to hypertension, OSA is associated with atrial fibrillation, coronary heart disease, stroke, and increased mortality [10-13]. Second, hypertension is a well-known major risk factor for the development of CVD and carries substantial importance for cardiovascular-related outcomes. While OSA and hypertension are associated with negative health outcomes, risks for these disorders are modifiable. Current risk prediction for OSA relies on questionnaire data, and hypertension risk requires a clinician’s assessment or risk calculators. In REFRESH, OSA risk is measured using the Berlin questionnaire [14], which is commonly used in the clinical setting to screen for possible OSA. In addition, data captured by the Berlin survey includes self-reported hypertension status. Sleep variability has been associated with poor outcomes, including mortality, and could be an important modifiable risk factor for multiple disorders.

While tools currently exist to assess hypertension and risk for OSA, these data are not regularly collected in the context of objectively measured longitudinal sleep. Using the framework that both OSA and hypertension are independently associated with CVD, we used the biometric data obtained in the REFRESH Study to determine if key sleep metrics, such as sleep duration, sleep variability, and social jet lag, are independently associated with OSA risk and self-reported hypertension. This study aims to examine whether longitudinal sleep patterns from activity trackers can predict risk of OSA and hypertension, as defined by self-report responses to the Berlin questionnaire.

## Methods

### Ethical Considerations

This study was approved by the Scripps Institutional Review Board (IRB# 21‐7863) and was registered on ClinicalTrials.gov as an observational study (NCT05197738). Informed consent was obtained through the REFRESH Study app, with several screens describing the details of the study as well as risks/benefits to the participants. Participants who signed the mobile consent were enrolled in the study. Participants had the option to exit the study at any time. All data collected after enrollment were deidentified, and all data transfers were integrated by CareEvolution via the study app and securely transferred to Scripps Research for storage on secure servers in an effort to ensure participant anonymity. Participation in the study was completely voluntary, with no compensation provided for any portion of the study. There was no cost to the subject for study participation.

### Study Design, Participants, and Recruitment

This was an observational cohort study with no a priori defined safety end points. The main outcome of interest was the development of an interactive platform that allows for collection of participant-provided data for the determination of health-related factors associated with sleep and different aspects of psychological, physical, and cognitive health. Data collection started in December 2021, and data presented here were collected through July 2024. The inclusion criteria were as follows: (1) adults ≥18 years of age; (2) current US resident; and (3) ability to utilize the REFRESH Study app on participant’s own smartphone device. The only exclusion criterion was the inability to consent. Recruitment occurred through unpaid posts on social media.

### REFRESH Survey Data Collection

**Figure 1.**
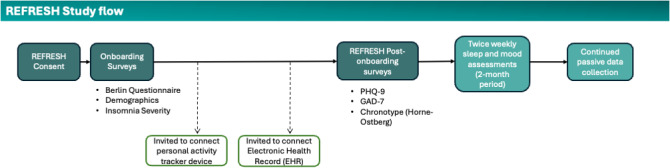
Study flow diagram. General outline of the participant’s engagement in the study. GAD-7: Generalized Anxiety Disorder 7-item; PHQ-9: Patient Health Questionnaire 9-item; REFRESH: Research Framework for Exploring Sleep Health.

Once the study participants completed the informed consent process, they completed the Berlin Questionnaire, which screens for OSA and contains a question to self-report hypertension, and it is a required survey for the study. Completion of the Berlin Questionnaire results in binarized OSA risk, specifically, low risk or high risk. Participants also provided demographic information detailing sex at birth, race and ethnicity, and socioeconomic status. Since the initiation of the study, the demographics questionnaire has been expanded to include other health data questions related to medical comorbidities; however, all participant data were analyzed and presented following the original demographic questionnaire. Additionally, participants reported on the use of prescription and over-the-counter sleep aids, as well as whether their main sleep was with a sleeping partner. Participants were asked to fill out the Insomnia Severity Index [15], the Patient Health Questionnaire–9-item [16], the Generalized Anxiety Disorder–7-item [17], and the Horne-Ostberg Morningness/Eveningness questionnaire [18]. Also, participants were invited to optionally connect their electronic health record to the app. During the first 2 months of the study, participants received a push notification on 2 random days of the week to obtain assessments of mood, stress, and subjective sleep that day. Figure 1 shows a diagram of the study flow.

### REFRESH-Linked Activity Devices

Participants were given the option to connect their personal activity tracker device to provide sleep, activity, and heart rate data. Devices were not provided to the participants. If participants linked their personal device, data prior to the onset of the study was included, with the participant’s consent. While the study was compatible with Fitbit, Apple Watch, Garmin, and Oura devices, we elected to include only individuals who linked one type of device technology in this study (ie, Fitbit) due to the majority of our study population utilizing this specific DAT. Additionally, the existing sleep literature investigating DATs and passive data collection overwhelmingly employs Fitbits when examining sleep health. We intend to examine the additional DATs utilized in the REFRESH Study to continue assessing the predictive potential of these devices in health risk prediction and modification. Aside from DAT data, all data collection occurred through the REFRESH app.

### Sleep Data Parameters

Additionally, we imposed the following parameters to limit the noise and variability in the analyzed sleep data: (1) only utilized daily sleep data corresponding to the main (primary) sleep period and did not include data pertaining to short sleep periods (naps) throughout the day; (2) each participant had a minimum of 14 nonconsecutive days of sleep data and a maximum of 4 years of daily sleep data. We then obtained average sleep metrics (ie, efficiency, minutes asleep, minutes awake, sleep variability, and social jet lag) [19,20] per person, parcellated out by weekday or weekend timeframes. Sleep efficiency is defined as:


$$\frac{total\;sleep\;time}{total\;recording\;time}\times 100\%$$

Minutes asleep utilized minutes from the main sleep duration (as opposed to total sleep time over the entire 24-h period), and sleep variability, which measures the magnitude of day-to-day fluctuations in an individual’s nightly total sleep duration, is the standard deviation of minutes asleep [19]. Lastly, social jet lag is defined as the difference between the average weekend sleep duration and the average weekday sleep duration [20]. Fitbit metrics and survey responses were pulled directly from the MyDataHelps platform. Additional information on the Fitbit metrics is available for download via the MyDataHelps Designer Help Center platform, within the Fitbit Sleep Log Data Export Format subsection [21].

### Statistical Analyses

All data were analyzed using R version 4.4.0, including the tidyverse, lubridate, and car packages. Sociodemographic and self-report behavioral characteristics, including age, sex, race and ethnicity, educational attainment, household income, presence of a sleep partner, sleep medication use, chronotype, BMI, comorbidities (including hypertension), and OSA risk (via the Berlin questionnaire), along with Fitbit-derived metrics, including (1) weekend and (2) weekday sleep variability, (3) weekend and (4) weekday minutes awake, (5) weekend and (6) weekday minutes asleep, (7) sleep efficiency, (8) social jet lag, and (9) resting heart rate, were included in the analyses. Continuous variables were inspected for normality by examining skewness and kurtosis. Chi-squared and independent *t* tests were performed to examine differences in demographic and behavioral characteristics between participants with either low or high risk for OSA. Multicollinearity via variance inflation factor was examined across all independent Fitbit sleep metrics (ie, efficiency, minutes asleep, minutes awake, sleep variability, and social jet lag) against the binary outcome of OSA risk.

To understand the relationship between Fitbit sleep metrics and high OSA risk, as well as sleep metrics and self-reported hypertension, independent binomial logistic regression models utilizing Fitbit metrics, self-report behavioral characteristics, and common covariates were performed. Covariates tested included the following: age, sex, race and ethnicity, educational attainment, household income, presence of a sleep partner, sleep medication use, chronotype, BMI, and resting heart rate. Final logistic regression models only included covariates that demonstrated a statistically significant relationship with the outcomes of interest. Corresponding metrics, including odds ratio (ORs) for binomial regression models, are presented in the later tables.

Given the exploratory nature of the REFRESH study, and the intent to generate a large database including DAT data to explore relationships between many sleep-related outcomes, there was not an a priori power analysis or sample size calculation. Rather, the intent was for post hoc power to be reported alongside exploratory analyses to inform future, a priori calculations and study design. As such, for primary and secondary outcomes, post hoc power calculations were conducted using G*Power version 3.1.9.7 [22] to determine the achieved power, given the following assumptions: for logistic regression models, with a binary outcome (probability of null hypothesis=0.50), sample size=391, and α=.025 to account for multiple comparisons. ORs utilized for post hoc power calculations were those reported in the outcomes analyses.

## Results

### Overview

**Figure 2.**
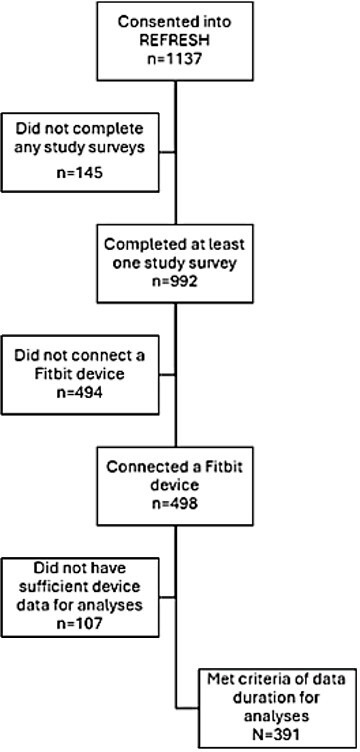
Recruitment and participant enrollment diagram. Outline of patient recruitment and flow diagram of individuals included in the analysis. REFRESH: Research Framework for Exploring Sleep Health.

Our sample of 391 adults predominantly comprised women (68%, 247/364) with a mean age of 48 (SD 13.62) years (Figure 2). The sample was predominantly White (65%, 231/353) followed by multiracial (17%, 61/353) and Hispanic or Latino participants (6.5%, 23/353), as outlined in Table 1.

**Table 1.** Demographic characteristics of REFRESH^a^ study participants (n=391).

Characteristics	Value, n (%)^b^
Age^c^ (y)	
Mean (SD)	48.14 (13.62)
Range	19-80
Sex^c^	
Male	117 (32.1)
Female	247 (67.9)
Race and ethnicity^c^	
White	231 (65.4)
Black	12 (3.4)
Asian	17 (4.8)
American Indian or Alaska Native	6 (1.7)
Hawaiian or Pacific Islander	1 (0.28)
Middle East and North Africa	2 (1)
Hispanic or Latino	23 (6.5)
Multiracial and Other	61 (17.3)
Education	
Less than high school	4 (1)
High school graduate or GED^d^	25 (6.4)
Some college	82 (21)
College graduate	95 (24.3)
Advanced degree	90 (23)
No response	95 (24.3)
Income	
0 to US $25,000	56 (14.3)
US $25,001 to US $50,000	41 (10.5)
US $50,001 to US $75,000	44 (11.3)
US $75,001 to US $100,000	45 (11.5)
US $100,001 to US $150,000	57 (14.6)
US $150,001+	87 (22.3)
No response	62 (15.9)
US region^c^	
West	85 (27.7)
Southwest	24 (7.8)
Midwest	88 (28.7)
Northeast	42 (13.7)
Southeast	68 (22.1)

a REFRESH: Research Framework for Exploring Sleep Health.

b %: proportion of participants.

c Nineteen participants were missing data indicating age; 27 participants were missing data indicating sex; 38 participants were missing data describing race and ethnicity; and 84 participants were missing region of residence data.

d GED: general educational development

The mean BMI of our sample was 27.52 (SD 5.89) kg/m^2^, with the majority of our sample classifying as normal weight (39.1%, 111/284), followed by overweight (30.6%, 87/284), and then obese (29.2%, 83/284) (see Table 2). Over a quarter of our sample (26.6%, 92/346) endorsed having high blood pressure, and almost half of our sample (48.1%, 179/372) was at high risk for OSA. Additionally, the majority of our sample (40.4%, 158/391) demonstrated an evening chronotype.

**Table 2.** Clinical and psychometric characteristics of REFRESH^a^ study participants (n=391).

Characteristics	Value, n (%)^b^
BMI^c^	
Mean (SD)	27.52 (5.89)
Range	16.97-46.06
Underweight, BMI<18.5	3 (1.1)
Normal weight, 18.5≤BMI≤24.9	111 (39.1)
Overweight, 25.0≤BMI≤29.9	87 (30.6)
Obese; BMI≥30	83 (29.2)
Hypertension^c^	
No	254 (73.4)
Yes	92 (26.6)
Morningness-Eveningness (ME) Chronotype Score	
Morning chronotype	57 (14.6)
Evening chronotype	158 (40.4)
No preference	79 (20.2)
Unknown	97 (24.8)
Insomnia Severity Index^c^	
Absence of insomnia	119 (33.7)
Subthreshold insomnia	135 (38.2)
Moderate insomnia	65 (18.4)
High insomnia	34 (9.6)
Berlin score^c^ (OSA^d^ risk)	
Low risk	193 (51.9)
High risk	179 (48.1)
Sleep partner^c^	
No	130 (36.5)
Yes	226 (63.5)
Sleep medication use^c^	
No	211 (59.3)
Yes	145 (40.7)
Nonprescription medications	77 (53.1)
Prescription medications	68 (46.9)

a REFRESH: Research Framework for Exploring Sleep Health.

b %: proportion of participants.

c 107 participants were missing data indicating height in meters or weight in kilograms; 45 participants were missing data indicating self-report hypertension; 38 participants were missing data addressing insomnia severity; 19 participants were missing data addressing obstructive sleep apnea risk; and 35 participants were missing data indicating whether or not sleep occurred next to a partner, as well as sleep medication usage.

d OSA: obstructive sleep apnea.

The mean amount of sleep data per person was 2 years and 55 days of weekday sleep data and 2 years and 47 days of weekend sleep data, as outlined in Table 3. Sleep metrics from the 391 individuals yield a mean sleep duration of 6 hours and 25 minutes (SD 59 min) during the week and 6 hours and 31 minutes (SD 59 min) on the weekend (*P=*.16). Sleep variability was not significantly greater on the weekends (mean 94.93 min, SD 38.99 min) than during the weekdays (mean 90.49 min, SD 36.25 min; *P=*.10).

**Table 3.** Fitbit characteristics of REFRESH^a^ study participants (n=391).

Characteristics	Mean (SD)	Range
Days count		
Weekend	775.84 (502.64)	14 to 1460
Weekday	784.06 (500.14)	15 to 1460
Sleep efficiency (%) per day		
Weekend	85.86 (13.30)	43.67 to 100.00
Weekday	85.82 (13.44)	44.37 to 100.00
Minutes awake per day		
Weekend	55.50 (13.52)	0.00 to 94.64
Weekday	54.89 (13.53)	0.00 to 96.99
Minutes asleep per day		
Weekend	390.37 (58.63)	143.80 to 536.08
Weekday	384.40 (58.86)	141.08 to 613.95
Sleep variability per day		
Weekend	94.93 (38.99)	5.58 to 288.98
Weekday	90.49 (36.25)	25.82 to 226.83
Social jet lag	5.98 (33.07)	−308.20 to 119.94
Resting heart rate^b^	68.48 (8.91)	48.50 to 97.88
Tracker steps^b^	6781.32 (3909.95)	56.00 to 33,534.76

a REFRESH: Research Framework for Exploring Sleep Health.

b Six participants were missing data indicating resting heart rate; 4 participants were missing data indicating tracker steps.

Sleep variability, both on the weekends and weekdays, was significantly associated with OSA risk. Individuals with high OSA risk demonstrated significantly greater weekend sleep variability (mean 102.40 min, SD 40.68 min) than individuals with low OSA risk (mean 85.50 min, SD 32.76 min; *P*<.001). Similarly, individuals with high OSA risk demonstrated significantly greater weekday sleep variability (mean 98.89 min, SD 39.58 min) than individuals with low OSA risk (mean 81.46 min, SD 30.01 min; *P*<.001).

Conversely, sleep variability, both on the weekends and weekdays, was not significantly associated with self-reported hypertension. Individuals with self-reported hypertension did not demonstrate significantly different weekend sleep variability (mean 97.47 min, SD 43.18 min) than individuals not endorsing hypertension (mean 92.22 min, SD 35.97 min; *P*=.30). Similarly, individuals with self-reported hypertension did not demonstrate significantly different weekday sleep variability (mean 94.19 min, SD 38.75 min) than individuals not endorsing hypertension (mean 87.49 min, SD 34.06 min; *P*=.14).

Multicollinearity via variance inflation factor was not observed when separating the sleep metrics by weekend or weekday collection, nor was it observed when examining all the independent weekday sleep metrics together against OSA risk (ie, low or high risk) as the outcome. Likewise, multicollinearity of predictor variables was not observed when examining all the weekend sleep metrics together against the OSA risk outcome. Tested in independent single-predictor binomial logistic regression models, the following sociodemographic, self-report behavioral, and device-derived covariates were significantly associated with OSA risk: household income, sleep medication use, BMI, chronotype, and resting heart rate. As such, these covariates were included in the initial regression models examining significant associations with Fitbit-derived sleep metrics and OSA risk. Age, sex, and BMI as covariates were included in the final logistic regression models, as the inclusion of the remaining covariates did not improve model performance. Only BMI as a covariate was significantly associated with the clinical outcome of OSA risk (ie, low or high risk) based on the Berlin questionnaire, whereas all three covariates (age, sex, and BMI) were significantly associated with self-reported hypertension in the final models.

### OSA Risk and Fitbit Sleep Metrics

Holding BMI at fixed value, the odds of having high OSA risk increased by 159% for every 1-hour increase in weekday sleep variability (OR 2.592, 95% CI 1.613-4.400; *P*<.001). Holding weekday sleep variability at a fixed value, the odds of having high OSA risk increased by 22% for each unit (kg/m^2^) increase in BMI (OR 1.217, 95% CI 1.153-1.293; *P*<.001). The statistically significant association and strength of the association between high OSA risk and weekday sleep variability remained even with the inclusion of weekday sleep duration as an additional covariate; however, weekday sleep duration was not significantly associated with OSA risk (ie, low or high risk; *P=*.30).

Similarly, for weekend sleep, the odds of high OSA risk are increased by 93% for 1-hour increase in weekend sleep variability, while holding BMI at fixed value (OR 1.928, 95% CI 1.197-3.094; *P=*.006). As with weekday sleep variability, the odds of high OSA risk are increased by 22% for each unit (kg/m^2^) increase in BMI (OR 1.217, 95% CI 1.153-1.293; *P*<.001) while holding weekend sleep variability at a fixed value. The statistically significant association and strength of the association between OSA risk and weekend sleep variability remained even with the inclusion of weekend sleep duration as an additional covariate; however, weekday sleep duration was not significantly associated with OSA risk (ie, low or high risk; *P=*.13) (see Table 4). Post hoc power calculations for the main effect of sleep variability revealed 85% power for weekend and 99% power for weekday sleep variability predicting high OSA risk.

**Table 4.** Binomial logistic regression of sleep variability and OSA^a^ risk and self-reported hypertension^b^.

	Weekend			Weekday		
Outcome	B^c^ (SE)	*P* value	OR^d^ (95% CI)	B (SE)	*P* value	OR (95% CI)
OSA risk						
Sleep variability (h)	0.01 (0.004)	.01	1.928 (1.197-3.094)	0.02 (0.004)	<.001	2.592 (1.613-4.400)
BMI	0.2 (0.03)	<.001	1.217 (1.153-1.293)	0.2 (0.03)	<.001	1.217 (1.153-1.293)
Hypertension						
Sleep variability (h)	0.01 (0.004)	.09	1.520 (0.942-2.443)	0.01 (0.004)	.03	1.712 (1.062-2.917)
Age	0.02 (0.012)	.11	1.018 (0.996-1.042)	0.02 (0.012)	.10	1.019 (0.997-1.042)
Sex (female)	−0.66 (0.32)	.04	0.516 (0.273-0.978)	−0.69 (0.33)	.03	0.502 (0.264-0.953)
BMI	0.08 (0.03)	.003	1.078 (1.027-1.134)	0.07 (0.03)	.003	1.077 (1.025-1.132)

a OSA: obstructive sleep apnea risk derived from Berlin Score.

b Covariates included are BMI when assessing OSA risk as an outcome and age, sex, and BMI when assessing self-reported hypertension as an outcome.

c B: beta coefficient.

d OR: odds ratio.

### Hypertension and Fitbit Sleep Metrics

Holding age, sex, and BMI at fixed values, the odds of endorsing hypertension increased by 71% for every 1-hour increase in weekday sleep variability (OR 1.712, 95% CI 1.062-2.917; *P=*.03). Holding weekday sleep variability, age, and BMI at fixed values, the odds of endorsing hypertension decreased by 50% for women in comparison to men (OR 0.502, 95% CI 0.264-0.953; *P=*.03). Holding weekday sleep variability, age, and sex at fixed values, the odds of endorsing hypertension are increased by 8% for each unit (kg/m^2^) increase in BMI (OR 1.077, 95% CI 1.025-1.132; *P=*.003). The statistically significant association and strength of the association between hypertension and weekday sleep variability remained even with the inclusion of weekday sleep duration as an additional covariate, the latter of which was not significantly associated with hypertension (*P=*.17).

Conversely, for weekend sleep, the odds of endorsing hypertension increased by 43% for a 1-hour increase in weekend sleep variability (OR 1.432, 95% CI 1.062-1.928; *P=*.04). Of note, covariates were tested in previous models and did not statistically improve model performance when examining weekend sleep variability’s association with self-reported hypertension; however, the inclusion of weekend sleep duration did improve model performance, albeit weekend sleep duration’s relationship with hypertension was not statistically significant (*P=*.06). Holding weekend sleep duration at a fixed value, the odds of endorsing hypertension are increased by 43% for a 1-hour increase in weekend sleep variability (OR 1.432, 95% CI 1.018-1.998; *P=*.04). Among our sample, weekend sleep variability’s statistically significant relationship with hypertension exists as a univariate relationship or in the presence of weekend sleep duration (see Table 4). While 8 distinct Fitbit sleep metrics were independently tested as predictor variables against the separate outcomes of OSA risk (ie, low or high) and self-reported hypertension, only weekend and weekday sleep variability were statistically significantly associated with either outcome. The following Fitbit sleep metrics independently tested as predictor variables were not statistically significantly associated with either outcome: (1) weekend and (2) weekday minutes awake, (3) weekend and (4) weekday minutes asleep, (5) sleep efficiency, and (6) social jet lag. Despite significant findings, post hoc power calculations for the main effect of sleep variability revealed 42% power for weekend and 64% power for weekday sleep variability predicting hypertension.

## Discussion

### Main Article Findings

Our results show a robust association between increased sleep variability and heightened risk for both OSA and self-reported hypertension, independent of average sleep duration. These data demonstrate that biometrics from digital devices could have utility in predicting disease risk, thus making devices with similar capabilities a potential tool with clinical relevance in the future. Furthermore, we show that the decentralized digital studies such as REFRESH can uncover distinct and clinically meaningful predictive patterns in longitudinal biometric assessments. While the study question here focused on the relationship between the biometric data and two important human health outcomes related to CVD, there are multiple other health relationships that could be studied in a similar fashion.

### Sleep Variability and Health Outcomes

Using passively measured sleep data from DATs, we found statistically significant and independent associations with sleep variability and high OSA risk, as well as self-reported hypertension. Specifically, our measurements showed that a 1-hour increase in sleep variability was associated with a more than 2-fold increase in having high OSA risk. Similarly, the odds of self-reporting hypertension were increased by almost 2-fold for every 1-hour increase in sleep variability. For context, this cohort had an average sleep variability of approximately 1.5 hours. While the data that we present here does not mechanistically link sleep variability with high OSA risk or hypertension, others have demonstrated evidence that supports our findings of a connection of sleep variability to both high OSA risk and hypertension. For example, Lechat et al [23] showed that higher night-to-night variability in OSA severity, suggesting variability in nightly sleep, had a larger association with uncontrolled hypertension compared to those with less variability in OSA severity. Furthermore, Xu et al [24] showed that 1 hour of increased sleep variability decreased blood pressure dipping (absent blood pressure dipping is associated with negative outcomes) by 1.39%. Mechanistically, sleep variability is associated with blunted diurnal cortisol slopes [[25]], which are associated with hypertension [26]. Inflammation may also play a role as sleep irregularity is associated with increased inflammatory markers [27,28], which are elevated in those with hypertension [29], with some data showing that inflammation occurs prior to the development of hypertension [30].

Sleep variability has also been shown to be important for CVD beyond risk for OSA and hypertension, highlighting the importance of sleep variability in the context of human health. For example, using a data set of over 100,000 Fitbit users with limited health outcomes data, our group previously reported on the association of sleep duration and sleep variability with obesity, with both short sleep duration and increased sleep variability associating with increased obesity (using self-reported height and weight data) [31]. Other studies suggest that sleep variability is a novel risk factor for coronary atherosclerosis *independent* of sleep duration and quality [32,33]. Furthermore, a 2023 UK Biobank study showed that sleep irregularity, and thus, more sleep variability, was a stronger predictor of all-cause mortality than sleep duration [34].

The American Heart Association recently added sleep health to their Life’s Simple 7 lifestyle recommendations (with components of smoking, obesity, physical activity, healthy diet, cholesterol, blood pressure, and fasting glucose), now dubbed Life’s Essential 8 [35]. Notably, the sleep recommendation in Life’s Essential 8 suggests focusing on a systematic assessment and inclusion of sleep duration to reflect sleep health within the construct of cardiovascular health; however, there are no recommendations beyond that of sleep duration within the context of CVD. Our study adds to the growing body of evidence that multiple aspects of sleep, beyond duration, are important for CVD-related outcomes. Sleep variability, and not just sleep duration, is an important component of human health.

### Decentralized Research and Digital Devices in the Context of Human Health

People are amassing important health-related biometric data on a population scale [36]. Using data from 2019 to 2020, Dhingra et al [37] estimated that over 70 million adults had used an activity tracker (eg, smartwatch or band, ring) in the prior 12 months [37]. While the range of wear time is wide, with one systematic review reporting a use range of 1 day to 59 months [38], there is a real opportunity to harness these biometric data to extract clinically relevant health information that could inform clinical decision making in the future. Activity trackers would be most useful clinically if they were able to provide information leading to disease diagnoses, though the technology has not yet become advanced enough to make this relevant beyond some specific conditions, such as atrial fibrillation. However, the ability to accurately predict risk for certain diseases—particularly where that risk is modifiable—such as OSA, hypertension, CVD, and type II diabetes mellitus would be of major benefit to patients as well as clinicians. There are multiple scores and algorithms used to measure clinical risk, which require the input of both subjective and objective data about the patient, including labs, vital signs, medical history, smoking history, and alcohol use. However, if digital devices, agnostic to other information about the patient, can objectively assess health risks for patients as well as our clinical scoring algorithms, then these data could become incorporated into clinical care and have the potential to better treat and sooner identify health risks. Individual biometrics could eventually play a role in clinical care to not only augment a personalized approach to sleep health but to address modifiable health factors in general.

### Limitations

This is a large prospective cohort study with data obtained from statistically meaningful associations. First, our cohort has a higher number of evening chronotypes than might be expected in the general population, but arguably, these adults could have the greatest impact from changes in sleep, given that evening chronotype is associated with obesity, diabetes, high blood pressure, and unhealthy diet habits. Second, having more women in the study compared to men could also be considered a limitation, though there is a general paucity in research on women’s health as it is. Third, this study was exploratory in nature and not statistically powered a priori. Rather, post hoc power was reported and determined to be adequate for predicting high OSA risk but not self-reported hypertension from DAT-derived sleep variability. Fourth, we also note that our OSA risk assessment (ie, low or high risk) and hypertension data were obtained from a subjective questionnaire and do not reflect a formal diagnosis of either disorder. While not a replacement for a formal diagnosis, using a validated clinical screener commonly used in the clinical setting affords real-world utility and generalizability in assessing risk for these two modifiable conditions. Future studies would benefit from examining the predictive potential between both formal OSA and hypertension diagnoses and sleep metrics passively derived from DATs. Fifth, our study likely selects for individuals who may have concerns about their own sleep, possibly due to poor sleep health, suggested by a large proportion of our study population (ie, 40.7%, 145/356) endorsing sleep medication use, including prescription and nonprescription sleep aids. However, having a cohort enriched with poor sleepers may have permitted a greater focus on the relationships this study has addressed. Finally, we elected to only include Fitbit-derived metrics due to the majority of our study population utilizing this specific DAT; however, we have no reason to believe our findings would not be generalizable to other DATs. Additionally, the existing sleep literature investigating DATs and passive data collection overwhelmingly employs Fitbits when examining sleep health, which aids to the generalizability and reproducibility of our findings. We aim to extend our models to other device types collected in the REFRESH study.

### Conclusions

Decentralized studies utilizing devices that people have already purchased have the potential to obtain sleep information from many individuals over a long period of time. Sleep variability may be an important target for risk modification of certain diseases, though the causality of the relationships found here has yet to be determined. Digital sleep health studies have the potential to uncover meaningful clinical relationships in a research context.

## Abbreviations

CVD: cardiovascular disease
DATs: digital activity trackers
OR: odds ratio
OSA: obstructive sleep apnea
REFRESH: Research Framework for Exploring Sleep Health
VIF: variance inflation factor
